# The First Meeting with Patients

**Published:** 1898-12

**Authors:** Dwight M. Clapp


### THE FIRST MEETING WITH PATIENTS.[2]

BY DWIGHT M. CLAPP, D.M.D.

Mr. President and Fellow-Members of the Harvard Dental Alumni,—Our friend Joe Jefferson gave an informal talk at the University Club not long ago, during which he was asked which he considered the more difficult to act, tragedy or comedy. He said he did not think he could answer the question better than to repeat the reply of a late English actor when asked the same question. He said, in substance, "Let me be never so weary and out of sorts, I can pull myself together enough to act tragedy at my best; but comedy,—ah, that is a serious business!"

To me it is a serious business to stand before so many Harvard men and talk about the first meeting with patients.

In 1868 or 1869 I had several large gold fillings put into my superior bicuspids by one of the brightest Boston operators of that time, a brilliant mechanic, but lacking in anatomical and collateral knowledge, such as is offered the students of the Harvard Dental School to-day. As a result, the pulps of these teeth were not properly protected, and much trouble followed.

While living in Geneva, in 1870, one or more of these teeth

---

[2] Read before the Harvard Dental Alumni, "Alumni Day," June 27, 1898.

died. It may have been the result of the shock caused by the declaration of war between France and Germany, but I always laid it to too close proximity of the fillings to the nerves of the teeth. I treated them myself as best I could, and some time afterwards the late Dr. Moffatt, whom many of us older ones remember with peculiar affection, treated one by piercing the alveolus at the point of the root.

With very slight interruptions, these four dead bicuspids have been perfectly comfortable for more than a quarter of a century, and are now doing perfect service, reinforced by gold caps. Naturally I have great respect for some so-called dead teeth, and these in particular.

Dreaming the other night, I seemed to experience a stab of pain in the alveolar region about these teeth. It gradually increased. There was heat, and the thumping throb preceding the abscess stage. Surely I must see a dentist.

Morning seemed to dawn. Shortly I was approaching the office-door of the village dentist. I noticed that the paint was dingy, a blind at the window hung by one hinge, and the corners of the steps were packed with accumulated dust and dirt. I opened the door, my heart seeming to occupy a greater portion of my mouth than did my teeth. One glance photographed the entire office on my mind. There was a hole in the carpet, the covering of the chair was ragged, the operating-case was strewn with dirty instruments, and a glass, partly filled with water, bore on its rim the blood-marked curve of the lips of the previous victim.

And my nose, it was besieged with the fumes of last night's pipe and cigar smoke, shaded down with the perfume arising from the belligerent bacteria inhabiting the cuspidor.

I was startled by a not over-gentle salutation,—"Well, how are you?" "Can you tell me," I meekly asked, "if the doctor is in?" "Yes, I'm him. What's the matter with you?" I began telling him my troubles. "Oh, dead teeth, is it? Yer can't do nothin' with 'em. The only thing is to pull 'em out, and have in one of my plates adjusted to heat and cold. Got 'em fixed now with my new process, so that they are just as good whether the mercury is down to zero or whether she's a stickin' right up through the top of the glass. Great thing, I tell you!"

"But," I suggested, "these teeth have been dead a long time, and have not troubled me." "Troublin' you now, ain't they? I tell you there can't be nothin' done for dead teeth but to pull 'em out. Never was one but would have been better out than in.

You better set up here and let me yank 'em right out, and have it over with. You never will get no comfort until you have one of my plates, adjusted to heat and cold."

Just then the pugilistic bacteria got in another round, and I mustered up sufficient courage to feebly say that I thought I would try them until afternoon, and, if they were no better then, I might have them out. As I stepped out, half expecting to be forced back by some invisible power, I heard, as the door closed behind me, "Nothin' like that plate of mine, perfectly adjusted to heat and cold."

On reaching the street, I was reminded by another twinge that my troubles were not over. "Why not consult Dr. Hifalutin?" some one suggested, so I at once sought his office. On being ushered into the doctor's presence, I gave him a history of my troubles. He listened in grave silence, and, as I proceeded, I could see that he considered my case a desperate one, and that he must brace himself, so to speak, in order to do it full justice.

After I had finished the details, he majestically waved me towards the operating-chair, and said if I would be seated he would try to ascertain, by concise observation, if the condition of the affected parts gave pathological evidence corresponding to the train of symptoms I had narrated.

As he raised my lip and caught sight of my teeth and gums, his face darkened, and I could see that he considered the situation exceedingly complicated. "Yes," he said, "I fear the organs have lost their vitality; sometimes, however, great pain is exhibited in the bicuspid region when there is really no lesion there, the real cause being located in some one of the dentes sapientiæ. In your case, the appearance of the gingival borders would indicate stasis. The alveolar ridges may have become denticulated from the inflammatory action, causing the hypertrophy of the membranes and great preponderance of the circulatory fluids. Sometimes these cases, if not properly diagnosed and skilfully treated, lead to the most serious results. I had a case, the other day, that started from the same cause as this, where the osseous tissues involved included not only the alveolar plates, external and internal, the maxillary bone, and the superior and inferior turbinate, but also, I suspected, a slight affection of the extreme end of the nasal. These teeth had been deprived of their central nutritive organs only twenty-six and a half years, instead of twenty-eight, as yours have. The effect on the soft tissues was much more apparent to ocular interpretation. The mucous and submucous membranes were much distended. The masseter, external and internal pterygoid muscles were somewhat rigid, the stylo- and hyo-glossus more or less constricted, while the condition of the orbicularis oris and the levator labii superioris alæque nasi was such as to give a most strained and interesting appearance, from a purely scientific view, to the mouth on that side."

Cold perspiration was beginning to start from every pore.

"It is exceedingly fortunate for you that you came to some one able to diagnose and scientifically treat such a rare and complicated pathological condition.

"It will be necessary, I fear," he continued, "to trephine the external alveolar plate over the buccal root of the first bicuspid, curette the tract of suppuration, and insert a canula to insure perfect drainage for the broken-down and disorganized products of decomposition during the process of granulation and repair."

Drawing a long breath, and pulling myself together as best I could, I managed to faintly articulate that, if what he had said should strike my cerebrum with sufficient force to wake it up, and if the result should be a differentiation of his diagnosis and his prognosis, and if my medulla oblongata should insist upon it, I would return in the post-meridian to be operated upon.

Again I found myself in the street, discouraged and at a loss which way to turn for help. Suddenly raising my eyes to an attractive house, I saw, in modest letters, the name John Smith, D.M.D. I was filled with hope at once. Surely, *John Smith, D.M.D.*, must be able to aid me. Without hesitation I entered his neat apartment. Here, as at the other places, the whole surroundings were stamped on my mind in an instant. There was no display of wealth, but neatness and artistic harmony everywhere. It was homelike; there were no guillotines hanging from the windows or ceiling, no electrocuting appliances boldly displayed. There was no show of steel to further excite my already high-strung nerves, and no smell of ether to suggest that I must take an anæsthetic, or carbolic to indicate the sterilizing of scalpels and gouges. Everything was just nice.

A cheery voice said, "Good-morning." Turning, I saw a gentleman. Gentleman was stamped on form, feature, and bearing.

I again told my story, this time into a willing and sympathetic ear. The interested, thoughtful, and pleasant face of the doctor invited confidence. "Surely," he said, "teeth that have served you so long and well shall have our best efforts to keep and preserve them useful for a long time yet."

With a snow-white napkin and dainty fingers he lifted my lip to make the examination. With great care and minuteness he noted every feature of the case.

"I do not think we need expect any serious trouble," he said. "There has been some disturbance of the circulation about these teeth, but I hope it will be of short duration. I will make an application calculated to allay the inflammation, and you may hold cold water in your mouth or apply ice to your face to keep the blood away from the part as much as possible. If this fails, after a few hours' trial, you must come back. There are many other things I can do to promote a speedy return to health."

My fears allayed, there was immediately a lessening of the blood tension, and an improved condition which always follows when one has had an interview with his physician, in whose skill and honesty he has entire confidence.

I awoke with the feeling that I had found a dentist in whose hands I could safely trust my teeth; one whom it would be a satisfaction to have as my confidential dental physician, and a worthy associate wherever we might chance to meet.

